# Development of a Native *Escherichia coli* Induction System for Ionic Liquid Tolerance

**DOI:** 10.1371/journal.pone.0101115

**Published:** 2014-07-01

**Authors:** Marijke Frederix, Kimmo Hütter, Jessica Leu, Tanveer S. Batth, William J. Turner, Thomas L. Rüegg, Harvey W. Blanch, Blake A. Simmons, Paul D. Adams, Jay D. Keasling, Michael P. Thelen, Mary J. Dunlop, Christopher J. Petzold, Aindrila Mukhopadhyay

**Affiliations:** 1 Joint BioEnergy Institute, Emeryville, California, United States of America; 2 Physical Biosciences Division, Lawrence Berkeley National Laboratory, Berkeley, California, United States of America; 3 School of Engineering, University of Vermont, Burlington, Vermont, United States of America; 4 Botanical Institute, University of Basel, Basel, Switzerland; 5 Biology and Biotechnology Division, Physical and Life Sciences Directorate, Lawrence Livermore National Laboratory, Livermore, California, United States of America; 6 Department of Chemistry, College of Chemistry, University of California, Berkeley, California, United States of America; 7 Department of Chemical & Biomolecular Engineering, College of Chemistry, University of California, Berkeley, California, United States of America; 8 Biological and Materials Science Center, Sandia National Laboratories, Livermore, California, United States of America; Louisiana State University and A & M College, United States of America

## Abstract

The ability to solubilize lignocellulose makes certain ionic liquids (ILs) very effective reagents for pretreating biomass prior to its saccharification for biofuel fermentation. However, residual IL in the aqueous sugar solution can inhibit the growth and function of biofuel-producing microorganisms. In *E. coli* this toxicity can be partially overcome by the heterologous expression of an IL efflux pump encoded by *eilA* from *Enterobacter lignolyticus*. In the present work, we used microarray analysis to identify native *E. coli* IL-inducible promoters and develop control systems for regulating *eilA* gene expression. Three candidate promoters, P*marR’,* P*ydfO’,* and P*ydfA’*, were selected and compared to the IPTG-inducible P*lacUV5* system for controlling expression of *eilA*. The P*ydfA’* and P*marR’* based systems are as effective as P*lacUV5* in their ability to rescue *E. coli* from typically toxic levels of IL, thereby eliminating the need to use an IPTG-based system for such tolerance engineering. We present a mechanistic model indicating that inducible control systems reduce target gene expression when IL levels are low. Selected-reaction monitoring mass spectrometry analysis revealed that at high IL concentrations EilA protein levels were significantly elevated under the control of P*ydfA’* and P*marR’* in comparison to the other promoters. Further, in a pooled culture competition designed to determine fitness, the strain containing pP*marR’-eilA* outcompeted strains with other promoter constructs, most significantly at IL concentrations above 150 mM. These results indicate that native promoters such as P*marR’* can provide effective systems for regulating the expression of heterologous genes in host engineering and simplify the development of industrially useful strains.

## Introduction

The polysaccharides present in lignocellulosic biomass provide an attractive raw material for the production of renewable biofuels. Pretreatment of the biomass, e.g. via exposure to dilute acids at high temperatures or ammonia fiber expansion [1], [2], [3], [4], is necessary prior to saccharification of the material. Recently, hydrophilic ionic liquids (ILs) have emerged as pretreatment solvents because they are highly effective at solubilizing polysaccharides [5], [6], [7], [8]. To minimize the costs associated with washing pretreated biomass, and recycling ILs, a residual level (0.2–5wt/vol%) of IL typically remains in downstream stages of an industrial scale production process [9]. However, these low levels of IL, contaminating the sugar stream used for cultivation, are toxic to biofuel-producing microorganisms [10], [11], [12], [13]. Residual IL may eventually be utilized in an industrial set-up, in combination with host organisms engineered for IL tolerance, to prevent contamination of the cultures.

The natural IL resistance of a rainforest bacterium, *Enterobacter lignolyticus*, has been investigated by transcriptome analysis, which revealed the differential expression of 688 genes in response to the IL 1-ethyl-3-methylimidazolium chloride ([C_2_mim]Cl) [14]. Significant increases occur in several genes encoding membrane transporters, including one of the most highly upregulated genes that encodes a member of the multidrug efflux pump of the Major Facilitator Superfamily (MFS). This gene, *eilA*, was independently identified by functional screening for IL tolerance using a fosmid library of genomic DNA from *E. lignolyticus* [15]. Heterologous expression of *eilA* in *E. coli*, either controlled by its native promoter or by P*lacUV5*, dramatically increases tolerance to [C_2_mim]Cl. A low level of IPTG (e.g. 10 µM) induction of P*lacUV5* is optimal for expression of the EilA pump, whereas higher levels are inhibitory to microbial growth [15]. A similar effect is also observed with the overexpression of other membrane proteins [16], [17].

Well-characterized induction systems like P*lacUV5* are useful in laboratory studies of genes and pathways, but they are not amenable for use in industrial processes because of the cost of inducing reagents. Another drawback of P*lacUV5* is the lack of flexible induction, since a given concentration of the inducer and time of induction must be selected in advance and remain constant throughout the cultivation period [18]. An engineered microbial host may include numerous pathways and related genes that each require fine control, placing a limit on the number of available externally induced systems.

Because residual concentrations of IL in the saccharified biomass solution are likely to vary from batch to batch, dynamic control of gene expression would provide more uniform regulation and robust cellular growth than that afforded by constitutive expression [15], [18]. In contrast to constitutive expression systems, which are optimal only under certain constant conditions, a condition-responsive expression system adjusts to the actual reaction environment. Such expression optimization may become particularly important when integrated with the expression of metabolic pathways or other tolerance mechanisms. One way of developing such a dynamic regulatory system is via a transcriptional regulator that induces the expression of the pump in the presence its substrate, as is often found in natural expression systems for efflux pumps [15], [19], [20]. To this end, we use transcriptomics and proteomics to determine the usefulness of *E. coli* IL-responsive promoters to drive expression of the heterologous export pump EilA.

## Results and Discussion

### Transcriptomic response *of E. coli* to [C_2_mim]Cl

**Figure 1:**
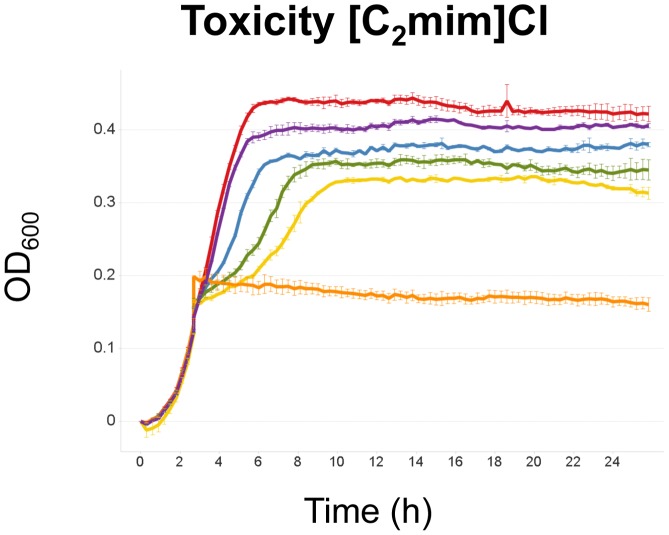
Toxicity of [C_2_mim]Cl to *E. coli* DH1 upon addition of [C_2_mim]Cl during exponential growth. Red: 0 mM [C_2_mim]Cl, purple: 50 mM [C_2_mim]Cl, blue: 100 mM [C_2_mim]Cl, green: 150 mM [C_2_mim]Cl, yellow: 200 mM [C_2_mim]Cl, orange: 400 mM [C_2_mim]Cl. Error bars represent standard errors.

The *E. coli* DH1 toxicity profile to [C_2_mim]Cl was determined by measuring the optical density of cultures after addition of the IL over a range concentrations (0–400 mM) in mid-log phase. [C_2_mim]Cl exposure resulted an increase in the lag phase and the doubling time (Figure 1). The impact on the lag phase and the doubling time was more pronounced when [C_2_mim]Cl was added in the culture at the time of inoculation (Figure S1). The transcriptional response of *E. coli* to [C_2_mim]Cl was determined by microarray analysis, 30 and 60 minutes after adding 150 mM [C_2_mim]Cl at mid-log phase (GSE51731). A sub-lethal concentration was chosen in order to reveal transcriptional changes that are specific to [C_2_mim]Cl rather than to general differences in the stage of growth. All transcripts with a differential expression greater than three fold are listed in Table S1. Results with a p-value>0.05 in the 30 min sample were not considered. In the samples collected 30 minutes after exposure, 122 genes were significantly upregulated and 66 genes significantly downregulated. After 60 minutes, a subset of these genes were no longer differentially expressed, suggesting that *E. coli* starts to adapt to [C_2_mim]Cl. For other genes the transcriptional changes persisted, as 94 genes remained upregulated and 46 genes remained downregulated. The transcriptional changes suggest a general stress response, as several of the induced genes (e.g. *ftnA, sodB, marRABC, pspABC, spy, degP*) have previously been reported to be involved in oxidative, solvent or salt stresses [21], [22], [23], [24], [25], [26].

### Characterization of selected [C_2_mim]Cl inducible promoters

To validate the microarray analysis, mRNA was prepared using conditions identical to the ones for the microarray analysis. Sixteen upregulated genes were chosen from the microarray dataset and their expression levels were evaluated by qPCR. Using this method, thirteen out of sixteen genes were confirmed to be significantly upregulated (Table 1). We chose three promoters, representing three different levels of induction, for further study: *ydfO* (high induction), *ydfA* (medium induction) and *marR* (low induction).

**Table 1:** Comparison of microarray and qPCR results for selected genes after stress with 150[C_2_mim]Cl.

	Microarray				qPCR
	30 min		60 min		30 min
Gene	Fold change	P-value	Fold change	P-value	Fold change qPCR ± st err
*ydfO*	12.53	0.04	8.6	0.04	116.61±21.81
*ydfA*	8.99	0.03	8.32	0.04	47.69±5.05
*marR*	4.12	0.02	4.26	0.02	17.37±3.10
*frmR*	13.24	0.01	12.11	0.02	43.58±6.89
*ybjJ*	3.71	0.01	3.36	0.02	3.17±0.18
*sodB*	7.43	0.01	8.44	0.02	15.85±4.93
*yoaB*	3.42	0.01	3.7	0.03	6.66±0.20
*rlmN*	4.48	0.01	4.08	0.02	2.43±0.46
*cysD* *	6.85	<0.01	3.34	0.02	0.63±0.15
*rsd* *	3.68	0.01	3.82	0.03	1.86±0.14
*ychF*	3.6	0.03	3.02	0.02	3.97±0.62
*degP*	5.33	0.03	4.91	0.04	5.38±1.10
*grxD*	4.57	0.01	4.73	0.02	11.53±0.38
*ybjG*	5.17	0.01	4.28	0.02	4.73±0.81
*htrL* *	3.57	0.04	3.31	0.04	1.03±0.63
*cueO*	4.45	0.02	4.04	0.04	13.93±2.76

Results are the average of three independent measurements. For microarray analysis, the corresponding p-value is given, for qPCR analysis the standard error is represented.

*: qPCR results do not agree with microarray results.

Of the genes corresponding to the promoters selected, *marR* is the most extensively studied. MarR is a transcriptional regulator that functions as an autorepressor [27] and is associated with regulating the expression of multidrug efflux systems, stress response systems, metabolic pathways and virulence factors [28], [29]. Further, the expression of *marR* is regulated by several transcriptional regulators involved in stress response, and includes MarA, Rob and SoxS [30], [31]. In contrast to MarR, very little is known about the primary function and regulation of *ydfO* and *ydfA*, both of which are predicted to encode genes of unknown function belonging to the Qin prophage. Interestingly, YdfO overexpression has been found to increase resistance of *E. coli* to oxidative stress [32].

**Figure 2:**
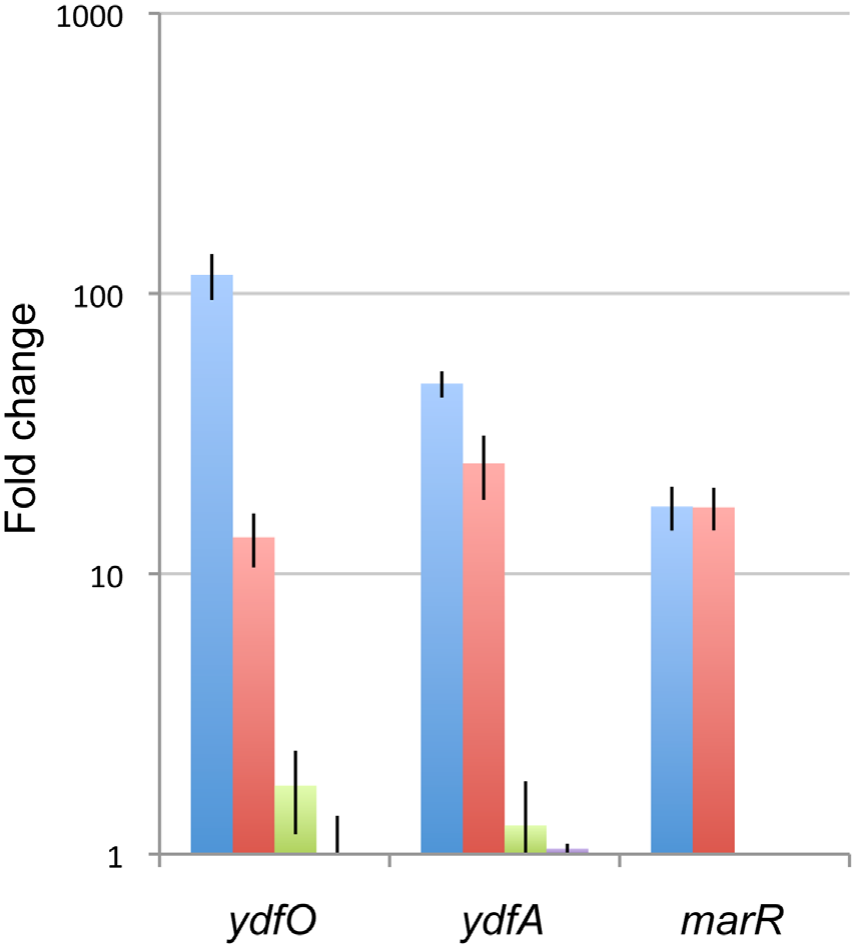
qPCR analysis of inducing P*ydfA’,* P*ydfO’* and P*marR’* in *E. coli* DH1 in 150 mM [C_2_mim]Cl, 100 mM [C_2_mim][CH_3_COO], 150 mM NaCl and 100 mM Na[CH_3_COO]. Cultures were grown until mid-exponential phase, when the compounds were added and RNA samples were collected after 30 min. The fold change on the chart represents the change in expression compared to an untreated control. *hcaT* was used as an endogenous reference to normalize the data. Error bars represent standard errors. Blue: 150 mM [C_2_mim]Cl, red: 100 mM [C_2_mim][CH_3_COO], green: 150 mM NaCl, purple: 100 mM Na[CH_3_COO].

To assess whether the upregulation of the selected promoters was specific to the presence of the [C_2_mim] cation rather than a general response to osmotic stress, the changes in expression of *ydfO, ydfA* and *marR* transcripts were measured in the presence of a similar IL, 1-ethyl-3-methylimidazolium acetate [C_2_mim][CH_3_COO] and the corresponding sodium salts of these ILs (Figure 2). Lower concentrations of the [C_2_mim][CH_3_COO] were selected to provide a comparable toxicity to [C_2_mim]Cl. The higher toxicity of [C_2_mim][CH_3_COO] may be due to the additive toxic effect of the acetate anion. A similar increased toxicity of [C_2_mim][CH_3_COO] compared to [C_2_mim]Cl has also previously been reported in *S. cerevisiae* [11]. All three genes were induced in 100 mM [C_2_mim][CH_3_COO], but not in 150 mM NaCl or 100 mM Na[CH_3_COO]. Therefore, the *ydfO, ydfA* and *marR* promoters appear to respond specifically to the presence of the [C_2_mim]^+^ cation.

**Figure 3:**
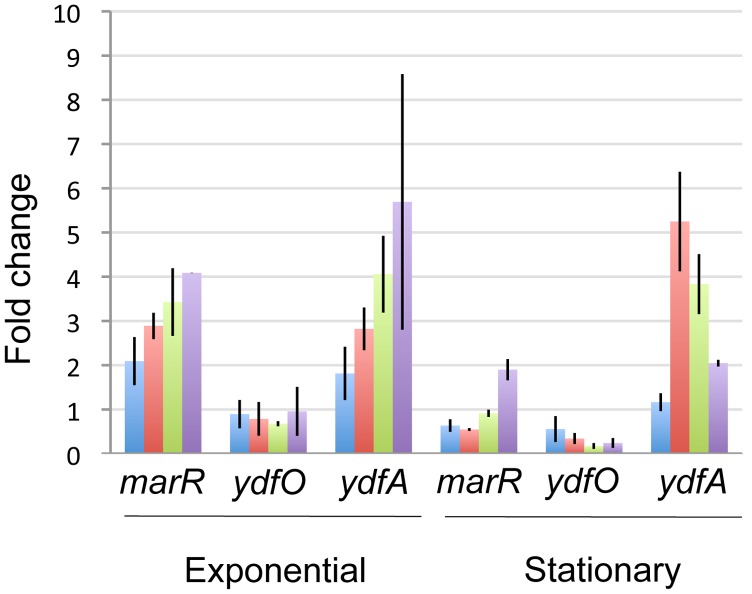
Transcript levels of *ydfO, ydfA* and *marR* during exponential and stationary growth after exposure to [C_2_mim]Cl. [C_2_mim]Cl was added from the beginning of growth at the indicated concentrations, and cultures were harvested during exponential growth (OD600 = 0.5) and stationary growth. The fold change represents the change in expression compared to an untreated control. *hcaT* was used as an endogenous reference to normalize the data. Error bars represent standard errors. Blue: 50 mM [C_2_mim]Cl, red: 100 mM [C_2_mim]Cl, green: 150 mM [C_2_mim]Cl, purple: 200 mM [C_2_mim]Cl.

Next, [C_2_mim]Cl was added at the beginning of growth and the expression levels of *ydfO, ydfA* and *marR* were determined by qPCR analysis at two time points (mid-exponential and stationary growth), in a range of 0–200 mM [C_2_mim]Cl (Figure 3). Under these growth conditions, which better represent anticipated IL levels present in fermenters, *ydfO* was no longer induced by [C_2_mim]Cl, but rather repressed in stationary phase at higher IL concentrations, indicating that the transcriptional response of the *ydfO* promoter is indeed growth phase dependent. *ydfA* and *marR* showed [C_2_mim]Cl dependent induction, and during exponential growth a clear dose dependence could be seen for both genes. In stationary phase, the induction pattern and promoter strength were very different. The inducibility of the *marR* promoter was reduced twofold, while the *ydfA* promoter became less inducible at higher concentrations of [C_2_mim]Cl (Figure 3).

### Evaluation of [C_2_mim]Cl responsive promoters to drive expression of *eilA*

It is known that overexpression of membrane proteins can lead to non-optimal cell growth [16]. To evaluate the growth burden of the EilA pump, culture density was measured as a function of increasing IPTG levels. In the growth conditions used in the present study, expression of the pump did not affect the lag phase and slightly reduced the maximum OD, however, it did impact the doubling time during mid and late exponential growth (Figure S2). Based on these results and other studies [15], 10 µM IPTG was used as the induction level in subsequent assays.

**Figure 4:**
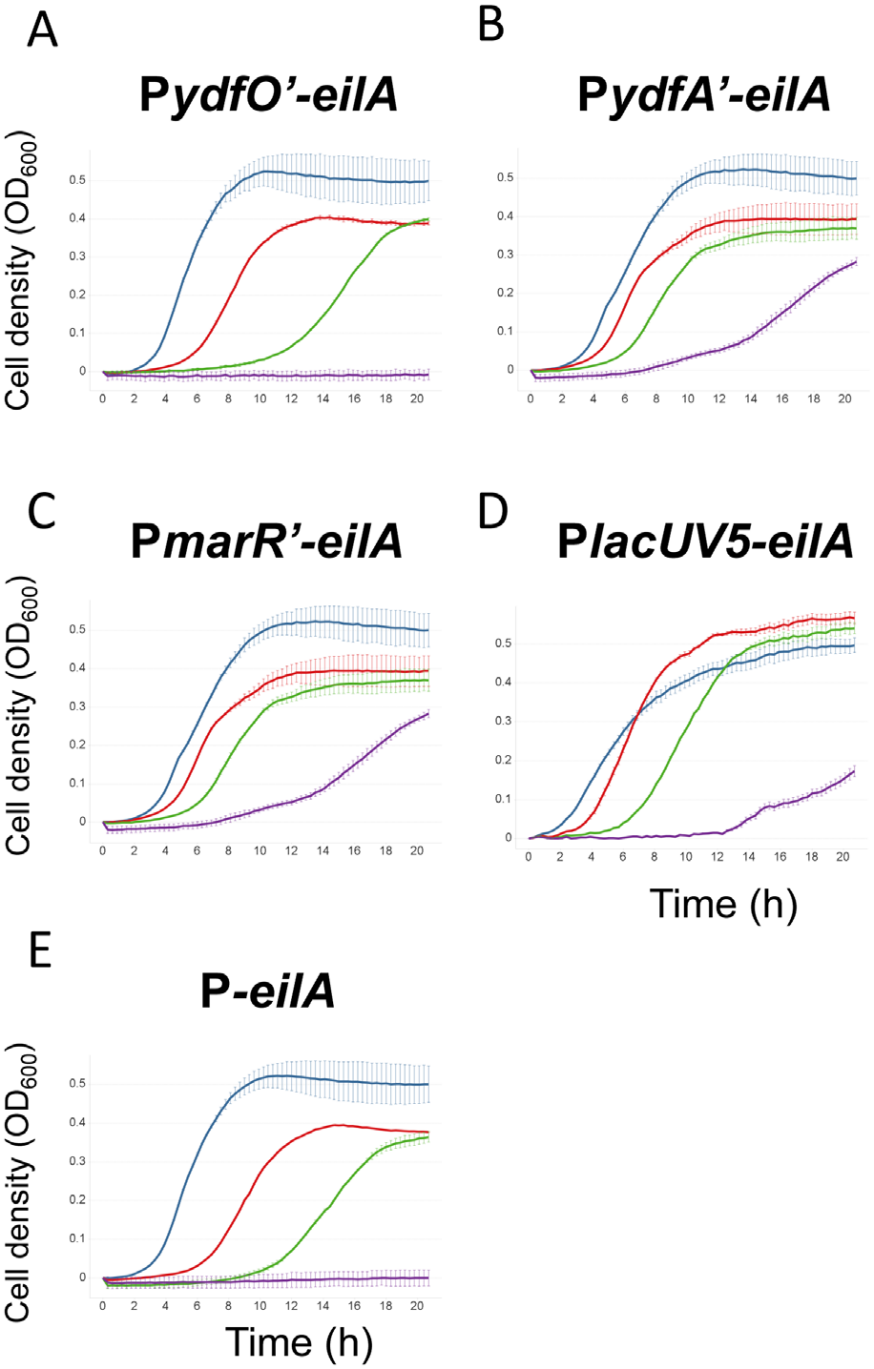
Growth of different *eilA* expression strains at increasing [C_2_mim]Cl concentrations. A–E: growth assays of *E. coli* DH10B carrying different promoter-*eilA* constructs. Due to day-to-day variability in the final cell density reached in the microtiter-plate experiments, growth curves were normalized to a start OD of 0 and a maximum OD of 0.5. A) pP*ydfO’*-*eilA*, B) pP*ydfA’-eilA*, C) pP*marR’-eilA*, D) pP*lacUV5-eilA,* E) pP-*eilA*. Error bars represent standard errors. Blue: 0 mM [C_2_mim]Cl, red: 100 mM [C_2_mim]Cl, green: 200 mM [C_2_mim]Cl, purple: 400 mM [C_2_mim]Cl.

To evaluate which of the [C_2_mim]Cl inducible promoters was best suited to drive the expression of the EilA pump, plasmid-borne *eilA* expression systems were constructed using the *ydfO, ydfA* and *marR* promoters and compared with the pP*lacUV5-eilA* construct induced with 10 µM IPTG in *E. coli* DH10B. A promoterless *eilA* construct (pP-*eilA*) served as the negative control. The resulting strains showed very similar lag, log and maximal growth under conditions without ILs (Figure 4).

Tested over a range of [C_2_mim]Cl concentrations (0 mM to 400 mM), the pP*ydfA’*-*eilA*, pP*marR’*-*eilA*, and pP*lacUV5*-*eilA* constructs performed similarly in conferring [C_2_mim]Cl resistance (Figure 4). As may be expected from the lack of inducibility of *ydfO*, in these test conditions (Figure 3), the pP*ydfO’-eilA* construct did not provide any significant resistance, and growth was similar to the strain carrying the promoterless pP-*eilA* construct.

**Figure 5:**
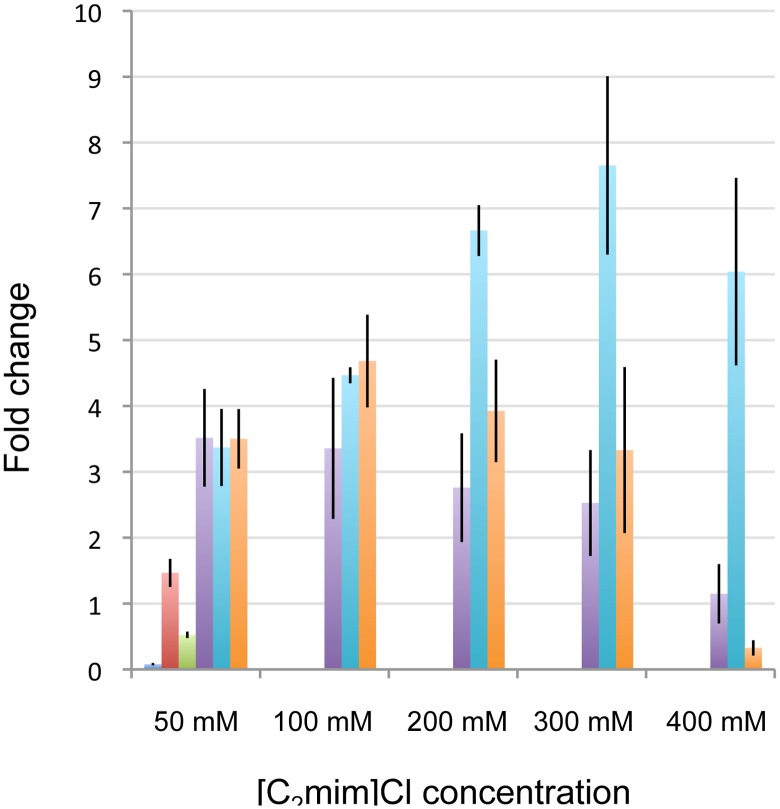
Relative abundance of *eilA* expression strains after competitive growth in pools with increasing [C_2_mim]Cl concentrations. Cultures were pooled and grown over 48-specific primers. 10 µM IPTG was added to induce the pP*lacUV5-eilA* construct. qPCR results were normalized using *cat* (the gene conferring chloramphenicol resistance on the expression plasmid) as endogenous control. Error bars represent standard errors. Dark blue: pP*lacUV5-rfp,* red: pP-*eilA,* green: pP*ydfO’-eilA,* purple: pP*ydfA’-eilA,* light blue: pP*marR’-eilA,* orange: pP*lacUV5-eilA.*

Since subtle differences in pump function or expression profile may result in a strain that has greater fitness [33], these strains were competed to establish which of the promoters was most suitable to drive the expression of *eilA*. For this, the different strains were pooled in equal proportions and grown in 0–400 mM [C_2_mim]Cl. For each IL concentration, the pooled cultures were grown for 48 h, during which they were subcultured six times into fresh medium, after which total plasmid DNA was isolated. qPCR analysis on the total plasmid DNA, using primers specific for the different constructs, allowed for quantification of the relative amounts of a specific strain in the pool (Figure 5). pP-*eilA* and pP*lacUV5-rfp* containing strains served as negative controls in the pool. The results of the competition assay (Figure 5) showed that the strain containing pP*ydfO-eilA* also disappeared from the pool, at even the lowest levels of [C_2_mim]Cl. At low concentrations (50 and 100 mM), pP*lacUV5-eilA*, pP*marR’-eilA* and pP*ydfA’-eilA* performed equally well, while at concentrations above 150 mM of [C_2_mim]Cl, pP*marR’-eilA* outperformed pP*lacUV5-eilA* and pP*ydfA’-eilA*. pP*ydfA’-eilA* outperformed pP*lacUV5-eilA* at 400 mM [C_2_mim]Cl (Figure 5).

### Expression levels of EilA

**Figure 6:**
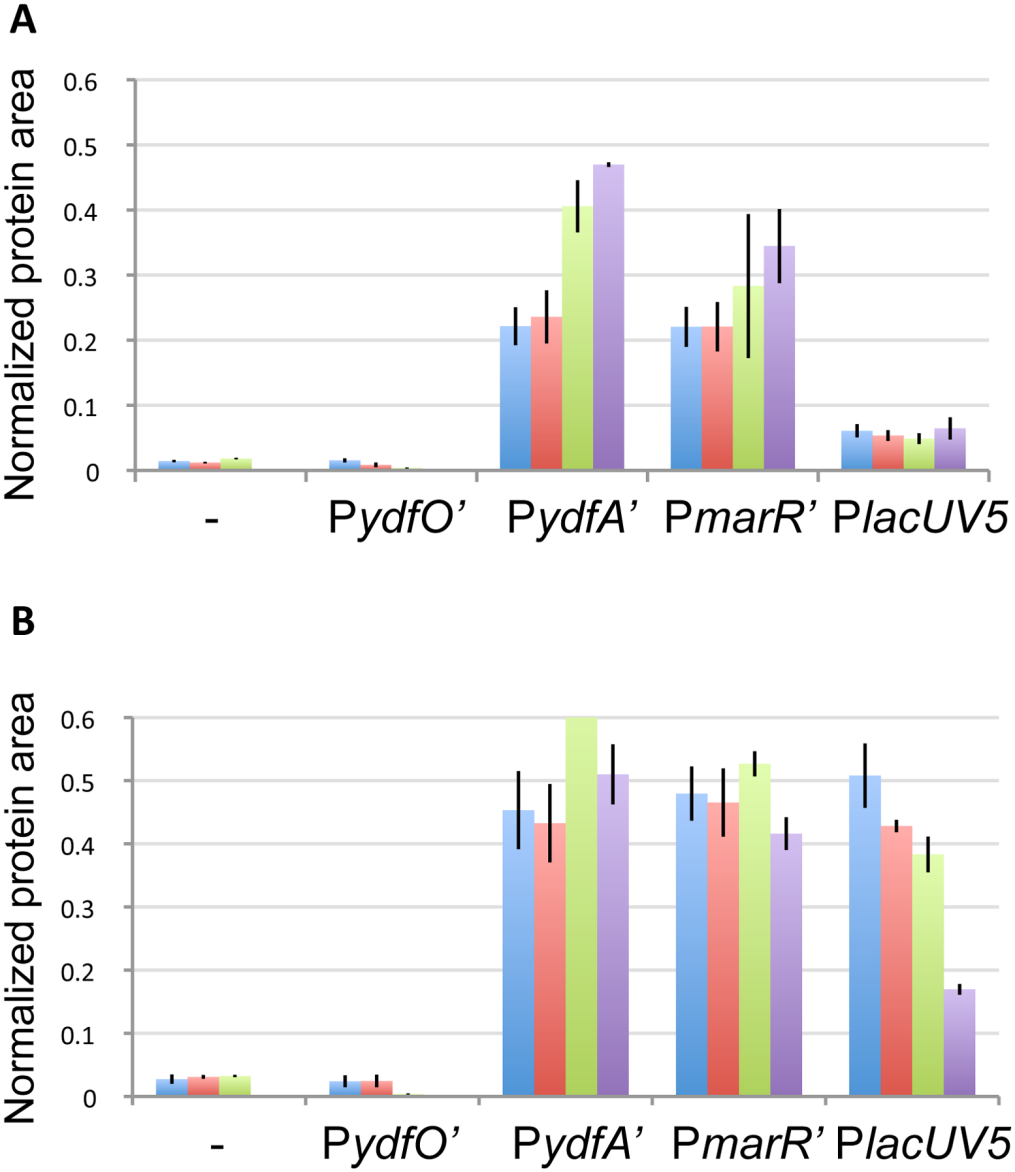
Quantification of EilA protein levels in different growth phases at increasing concentrations of [C_2_mim]Cl. Strains contain *eilA* expression constructs are as indicated in the graph. EilA protein levels were quantified during exponential (A) and stationary (B) growth, at the indicated [C_2_mim]Cl concentrations. EilA protein levels were normalized against Cat protein levels as an endogenous control. No growth was observed for pP*-eilA* and pP*ydfO’-eilA* in the presence of 400 mM [C_2_mim]Cl. Error bars represent standard errors. Blue: 0 mM [C_2_mim]Cl, red: 50 mM [C_2_mim]Cl, green: 150 mM [C_2_mim]Cl, purple: 400 mM [C_2_mim]Cl.

EilA protein levels in the different strains were determined by selected-reaction monitoring (SRM) mass spectrometry [34] and were normalized to the amount of chloramphenicol acetyl transferase (Cat) protein (expressed from the backbone of all the EilA expression plasmids) in the samples to correct for plasmid copy number variation (Figure 6). To examine the condition-responsive nature of the promoter activity, samples were collected at two time points (exponential and stationary growth) and a range of [C_2_mim]Cl concentrations (0 mM, 50 mM, 150 mM and 400 mM). No data were obtained for the pP-*eilA* and pP*ydfO’-eilA* strains at 400 mM [C_2_mim]Cl since no growth was observed, for these strains, in these conditions. Data for the remaining samples showed that the strains harboring pP*ydfO’eilA* and pP-*eilA* contain very low EilA levels resulting from basal promoter activity. The basal EilA levels resulting from pP*lacUV5-eilA* were significantly higher, but lower than those from pP*ydfA’-eilA* and pP*marR’-eilA*. The strains harboring pP*ydfO’eilA* and pP-*eilA* do not show IL-dependent induction in either growth phase. In contrast, the *ydfA* and *marR* promoters showed increased EilA levels at higher [C_2_mim]Cl concentrations during exponential growth. During stationary phase, no induction of EilA levels was detected at any of the [C_2_mim]Cl concentrations (Figure 6). The qPCR analysis had shown that the *ydfA* and *marR* promoters are inducible at this stage of growth (Figure 3) and a possible explanation is that the EilA accumulated in the cell membrane during exponential growth is sufficient to expel the IL, thus eliminating [C2mim]Cl inducibility of the *ydfA* and *marR* promoters in stationary phase. EilA expression from both these native promoters provides [C_2_mim]Cl tolerance in individually tested as well as mixed strain assays.

Differences in EilA levels were also seen for the P*lacUV5* expression system. During exponential phase 10 µM IPTG resulted in equal levels of EilA over different concentrations of [C_2_mim]Cl, but this was not the case during stationary growth, where a decrease in EilA levels with increasing [C_2_mim]Cl levels, was seen. These data suggest that the toxicity of [C_2_mim]Cl has a direct or indirect impact on P*lacUV5* function. While the mechanism of this decrease in promoter function is unclear, it might help explain the poorer performance of P*lacUV5* at higher [C_2_mim]Cl concentrations. Alternatively, the basal expression levels of EilA in the pP*lacUV5-eilA* strain may generally be lower than the pP*ydfA’-eilA* and pP*marR’eilA* strains in the absence of [C_2_mim]Cl.

### A model of EilA expression by different promoter systems

To provide insight into the results obtained with the various pump expression systems, we developed a mathematical model describing the dynamics of cell growth, substrate consumption, intracellular IL concentration, and pump expression. This model was used to compare the performance of the promoters that dynamically regulate pump expression in response to [C_2_mim]Cl (pP*ydfO’-eilA*, pP*ydfA’-eilA*, and pP*marR’-eilA*), a static expression system (pP*lacUV5-eilA*), and a negative control with no promoter (pP*-eilA*). The toxicity of ionic liquids has a major impact on cell viability, while pump overexpression shows a mild reduction in cell growth. Therefore, we included both of these terms as negatively impacting the growth of the culture, deriving model parameters from experimental data (Methods). The growth model is coupled with equations describing the diffusion and transport of intracellular ILs and pump expression dynamics.

**Figure 7:**
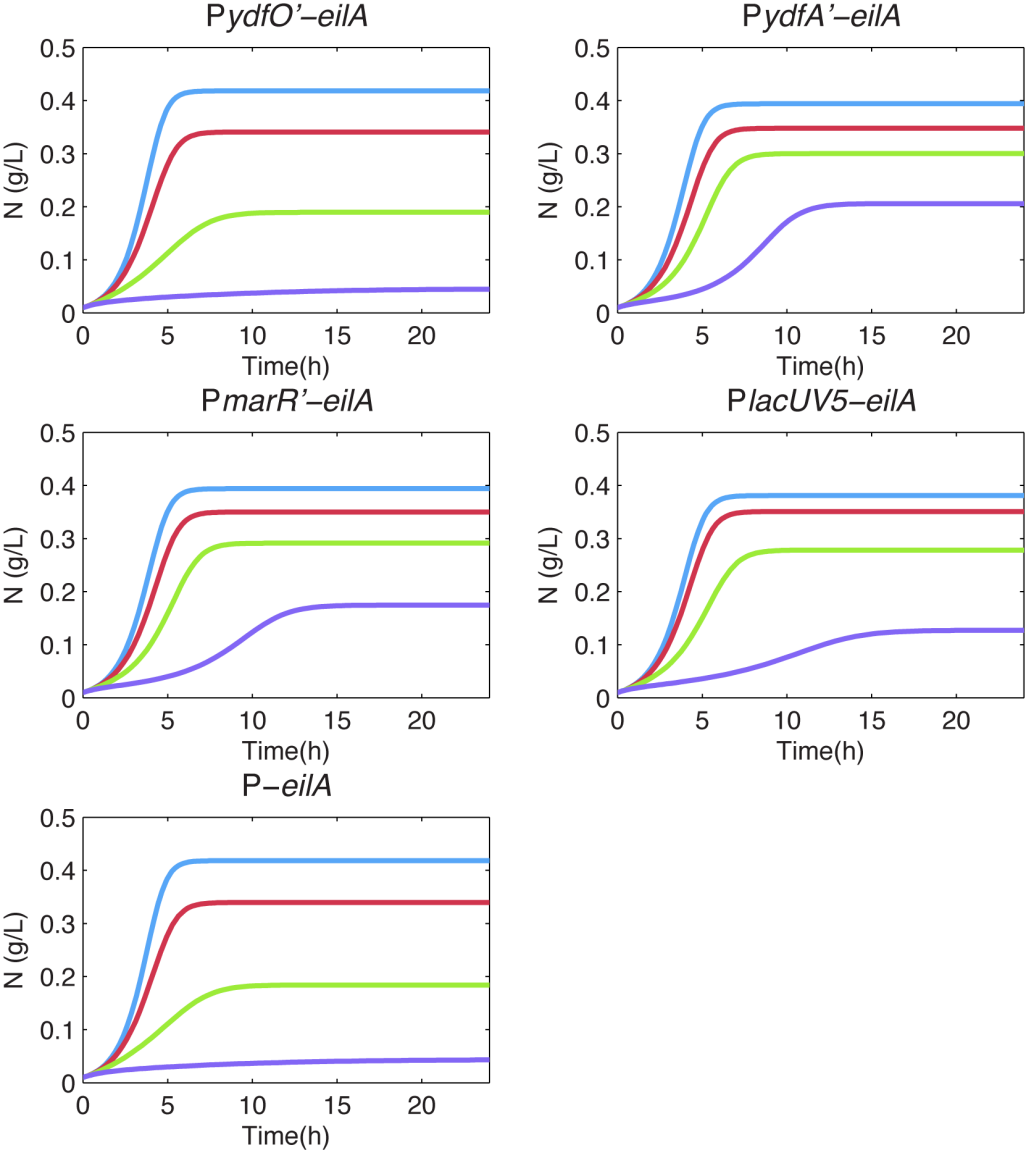
Modeling results comparing controllers at different [C_2_mim]Cl concentrations. A) pP*ydfO’-eilA,* B) pP*ydfA’-eilA*, C) pP*marR’-eilA*, D) pP*lacUV5-eilA*, and E) pP-*eilA*. Blue: 0 mM [C_2_mim]Cl, red: 100 mM [C_2_mim]Cl, green: 200 mM [C_2_mim]Cl, purple: 400 mM [C_2_mim]Cl.

The mathematical model indicates that P*marR’* and P*ydfA’* are the most responsive for a range of [C_2_mim]Cl concentrations, showing good agreement with experimental findings (Figures 4 and 7). In the absence of [C_2_mim]Cl, P*lacUV5* drives expression of the pump when it is not needed, reducing the growth rate. In contrast, condition-responsive induction allows the culture to grow to the maximum population density quite rapidly with less toxicity arising from pump expression. As the IL concentration is increased to an intermediate level of 200 mM [C_2_mim]Cl, P*marR’*, P*ydfA’*, and P*lacUV5* exhibit better regulation of pump expression than P*ydfO’* or no promoter. This result is due to the low levels of expression provided by the fully induced P*ydfO’* and the lack of promoter in pP*-eilA’*, both of which are insufficient to eliminate IL toxicity. At high concentrations (400 mM [C_2_mim]Cl), the P*marR’*, P*ydfA’*, and the IPTG-induced P*lacUV5* promoters enable growth, though P*lacUV5* is at a disadvantage relative to the others due to an increased lag phase and a decreased cell concentration during stationary phase. Therefore, P*marR’* and P*ydfA’* provide good performance by eliminating the burden of pump expression when IL levels are low and expressing pumps highly when IL levels are high. Subtle differences between these two promoters can be detected with sensitive competition assays, like those shown in Figure 5. These findings have practical implications, as residual IL concentrations are likely to vary from batch to batch. In such varying conditions, static pump expression systems like pP*lacUV5-eilA* will always be at a disadvantage relative to expression systems that respond to IL levels in a condition dependent manner. A pump expression system that responds to a changing environment will be robust to variations in production conditions.

### Conclusion

Promoters such as P*lacUV5* are commonly used in metabolic and host engineering proof-of-concept studies [40], [34]. However, developing economically-viable production systems for products such as biofuels restricts the use of expensive inducers such as IPTG. Further, the control provided by P*lacUV5* and other such commonly used systems may not be optimal for regulating mechanisms that are required for tolerance towards compounds whose levels vary from batch to batch or even during the course of a given culture. Examples of these compounds include inhibitors present in the carbon source from pretreated biomass [11], [35], [36], [37], [38] or accumulation of the biofuel product [34]. Dynamic control of gene expression may provide superior regulation of tolerance genes [17], [18], and studies have demonstrated that dynamic control of metabolic pathway genes can result in improved strain stability and production [39]. In the case of the EilA pump, an IL inducible system has also been established by using the pump associated *E. lignolyticus* repressor [21]. However, while tolerance genes can often function in heterologous hosts, it is less common to discover and deploy a corresponding heterologous regulatory system. In this study we used microarray data measuring transcript level responses to select three promoters that may allow condition-responsive control of *eilA*, P*ydfO’,* P*ydfA’* and P*marR’*. Other promoters that were part of our initial list may have also provided good candidates for the optimal expression of *eilA*. Alternate resources available for *E. coli* host engineering can also be utilized for promoter selection. For example the promoter library developed by Zaslaver and coworkers [40] can be used to test the promoters that respond to ILs. Since the post translational regulation of the tolerance pump is different from that of a reporter protein such as RFP or the native protein being controlled, each selection criteria presents its own caveats and strengths. However, as gene synthesis capabilities become less expensive a larger number of regulatory systems can be tested in order to select the most ideal or alternate candidates.

Despite the fact that the regulatory mechanisms underlying the induction of P*ydfO’,* P*ydfA’* and P*marR’* by [C_2_mim]Cl are unknown, we demonstrate the utility of these native host-organism promoters that are responsive to [C_2_mim]Cl, in developing control systems that provide condition-responsive control of target genes without the use of expensive reagents. Further, the use of orthogonal control systems may permit the integration of such tolerance mechanisms in strains with metabolic pathways with no conflict in control systems used. In this study, plasmid-based expression systems were used to demonstrate the concept of condition-dependent regulation of tolerance genes. For the development of an industrial host, strategies such as chromosomal integration may eliminate the need to use antibiotics, thus providing further avenues of optimization.

## Materials and Methods

### Bacterial strains, growth conditions and media

The *E. coli* strains used in this study were DH10B and DH1 (Invitrogen and ATCC33849). Bacteria were grown at 37°C in Luria-Bertani (LB) or M9 minimal medium (per liter: 200 ml 5×M9 salts, 2 ml 1 M MgSO_4_, 50 ml 20% glucose, 20 ml 5% casamino acids, 100 µl 0.5% thiamine, 100 µl 1 M CaCl_2_). Unless otherwise mentioned, all chemicals were purchased from Sigma-Aldrich (St. Louis, MO). Antibiotics were added as required to maintain plasmid selection. [C_2_mim]Cl (98%) was purchased from Sigma-Aldrich (St. Louis, MO) and [C_2_mim][CH_3_COO] (90%) was purchased from BASF (Ludwigshafen, Germany). Bacteria were adapted to M9 minimal medium by subculturing three times into fresh medium, after which they were stored as single-use glycerol stocks. For growth assays, strains were inoculated directly from frozen stocks into M9 minimal medium in 24-well plates and grown at 37°C in Tecan F200 or Tecan F200 pro microtiterplate readers (Maennedorf, Switzerland), measuring growth (OD 600) at 20 min intervals. For microarray analysis, strains were grown in 25 mL cultures in 250 mL flasks. For qPCR and proteomics analyses, strains were grown in 5 mL cultures tubes. For competitive growth experiments, 5 mL precultures were grown overnight in M9 minimal medium and pooled before the start of the competition assay.

### Construction of plasmids

The plasmids used in this study are listed in Table 2. *eilA* expression constructs were generated by circular polymerase extension cloning (CPEC) [41] by replacing the *lacI* repressor gene and the *lacUV5* promoter with the promoter regions of the *ydfO, ydfA* and *marR* genes. Primers used for amplification of vector and promoters are listed in Table S2.

**Table 2:** Plasmids used in this study.

Plasmids	Description	Reference
pP*lacUV5-eilA*	IPTG inducible *lacUV5* promoter driving expression of *eilA*, Cm^R^	[15]
pP*ydfO'-eilA*	promoter *ydfO* driving expression of *eilA*, Cm^R^	this work
pP*ydfA'-eilA*	promoter *ydfA* driving expression of *eilA*, Cm^R^	this work
pP*marR'-eilA*	promoter *marR* driving expression of *eilA*, Cm^R^	this work
pP*lacUV5-rfp*	IPTG inducible *lacUV5* promoter driving expression of *rfp*, Cm^R^	this work
pP-*eilA*	*eilA* without promoter, Cm^R^	[15]

### RNA isolation, microarray and qPCR analysis

For RNA extraction, cultures were collected in RNAlater (Qiagen, Hilden, Germany) to stabilize the RNA. Total RNA was isolated using the RNeasy Mini kit (Qiagen, Hilden, Germany). RNA quality and quantity were analyzed on 1% agarose gels and quantified using a NanoDrop ND-1000 spectrophotometer (Thermo Fisher, Waltham, MA). Labeling of RNA was performed as described previously [42], and the resulting cDNA was hybridized on commercially available *E. coli* K12 microarrays according to the manufacturers instructions (Nimblegen, Madison, WI). Microarray data analysis and normalization was performed using Arraystar (DNASTAR Inc., Madison, WI). To determine differentially expressed transcripts, an arbitrary cut-off of threefold change was used and genes with a p-value higher than 0.05 were omitted from the analysis. Microarray data have been deposited in the GEO database (GSE51731).

For qPCR analyses, purified RNA was additionally treated by Turbo DNase (Ambion Invitrogen, Carlsbad, CA), and reverse transcribed by Superscript III (Invitrogen, Grand Island, New York, USA) according to the manufacturers instructions. 1 µl of tenfold diluted cDNA was used as template in 20-µl reactions using Evagreen qPCR mix (Biorad, Hercules, CA). Primers used for amplification in the qPCR reaction are listed in Table S2. As endogenous controls *cat* (for competition experiments) or *hcaT* (other experiments [43]) were used and the fold change in expression of target genes was calculated by the 2-(ΔΔ-C(T)) method [44].

### Proteomics analysis

Cultures were grown for 18 h, and samples were processed as described in [34]. Relative protein production levels were determined by using targeted proteomics as described [34]. Samples were analyzed on an AB Sciex 5500Q-Trap mass spectrometer (AB Sciex Foster City, CA) operating in MRM-mode coupled to an Agilent 1100 HPLC (Agilent, Santa Clara, CA) operating in micro mode. SRM transitions and collision energies for the peptides were picked via the Skyline software [45]. Briefly, SRM selection excluded peptides containing cysteines, methionine and peptides with missed or repeating tryptic cleavage sites. Two peptides were selected for each protein with 2–3 y-series transitions per peptide. For analysis, 5 ug of each peptide digest was injected with 1 picomole of bovine serum albumin (BSA) as internal standard. Peptides were separated on a Agilent Zorbax 300 SB-C18 column (5 µm bead size, 150×0.5 mm) at 50 µl/min. The separation gradient was as follows: column was equilibrated with 98% A (2% Acetonitrile, 0.1% Formic acid) for 0.5 min and decreased to 65% A, 35% B (98% Acetonitrile, 0.1% Formic acid) over 7.5 min. The gradient was quickly ramped up to 90% B in one min and held at 90% B for 1.5 min after which it was ramped back down to 98% A in 1 min where it was held for 8.5 min to re-equilibrate the system for the next sample. Targeted proteomics data were analyzed using Skyline and quantification was based on the peak area for highest intensity transition for each peptide. All peak area integrations were performed by Skyline and were manually reviewed. Peak areas for each peptide were summed for corresponding protein and normalized to the sum of the Cat peak areas. Error bars represent the standard deviation of three biological replicates.

### Modeling

A system of ordinary differential equations was used to model the rate of change of biomass (N), substrate (S), pump (p), and intracellular IL (c_i_) concentrations.

Cell growth (Eq. 1) and substrate consumption (Eq. 2) were modeled using a modified form of the Monod equation [46]. The maximum growth rate (µ_max_), growth yield (γ), and half-saturation constant (K_s_) were selected to match the experimentally measured growth dynamics in the absence of [C_2_mim]Cl (Figure S3). The model includes inhibition by intracellular ILs and pump expression. The half-inhibition constant for IL, K_c_, and the Hill coefficient, h, were fit to the experimental data at stationary phase (Figure S4). The value of the half-maximum inhibition constant for pumps, K_p_, was set to match the data from Figure S2 for a range of relevant pump expression constants, α_p_ (Figure S5, Eq. 4).
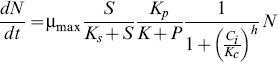
(1)

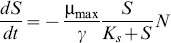
(2)


The equation used to model the rate of change of pump protein concentration depends on the promoter being modeled. For all promoter types there is basal expression, α_p0_, which was set to the exponential phase protein measurement without any IL present (Figure 6A). Also, in all cases, EilA proteins decay at the rate, β, which models both dilution due to cell growth and protein degradation. Thus, equation 3 represents protein production in the case of the promoterless construct, P*-eilA,* having only basal expression and degradation.
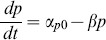
(3)


Equation 4 was used to model pump protein expression for the P*lacUV5* promoter. Along with the basal expression, this promoter has a constant protein expression level corresponding to the concentration of IPTG in the solution. This is represented by the constant, α_p_, which was set such that the total expression rate would equal the average of the protein measurements at the stationary phase (Figure 6B).
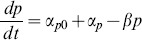
(4)


In the case of the dynamic controllers, P*ydfO’,* P*ydfA’,* and P*marR’,* the protein production rate depends on the intracellular IL concentration. Figure 6A indicates that the P*ydfA’* and P*marR’* promoters respond faster at higher concentrations of [C_2_mim]Cl due to the higher protein measurements in the exponential phase. Equation 5 represents the rate of change of pump protein for the dynamic controllers. Where the maximum (non-basal) protein production rate, α_p_, was set to the average of the protein measurements at stationary phase, and the half-maximum, γ_c_, was set to the concentration of IL that yields half of the maximum protein concentration during exponential growth (Figure 6A & B).
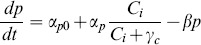
(5)


In order to model the intracellular IL concentration (c_i_), an IL mass balance was incorporated into the model to ensure that the total mass of IL in the reactor was constant and equal to the IL mass in the intracellular environment plus the IL mass in the extracellular environment. Passive diffusion of [C_2_mim]Cl through the cell membrane was modeled using Eq. 6.
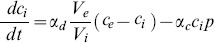
(6)


The gradient of IL concentration across the cell membrane is the driving force of this diffusion, and the concentration change is accounted for by the ratio between the extracellular and intracellular volumes. Intracellular volume was calculated by converting cell mass to number of cells and scaling by the volume of an *E. coli* cell. In all simulations IL is also actively exported from the cell at a rate proportional to the intracellular IL concentration and the pump protein concentration. The rate of export per pump protein, α_c_, was set to match experimental data (Figure 7). All model constants are given in Table 3. All simulations were performed in MATLAB R2012a (MathWorks) using the ode45 solver.

**Table 3:** Numerical constants used in simulations.

Symbol	Value	Description
µ_max_	1.7/h	Maximum growth rate
γ	0.041 g_cells_/g_substrate_	Growth yield
K_s_	8 g/L	Growth/substrate half-saturation constant
K_c_	0.06 M	IL toxicity half-saturation constant
H	2	IL toxicity Hill coefficient
K_p_	3	Pump toxicity half-saturation constant
α_d_	3.5(10^−6^)/h	Membrane permeability rate
α_c_	0.75/h-M	IL export rate
α_p0_	P*ydfo’-eilA*: 0.015/h	Basal protein expression rate
	P*ydfA’-eilA*: 0.22/h	
	P*marR’-eilA*: .22/h	
	P*lacUV5-eilA*: .06/h	
	P*-eilA*: .014/h	
α_p_	P*ydfo’-eilA*: 0.015/h	(Maximum) protein expression rate
	P*ydfA’-eilA*: 0.523/h	
	P*marR’-eilA*: .34/h	
	P*lacUV5-eilA*: .28/h	
β	1/h	Pump degradation coefficient
γ_c_	P*ydfo’-eilA*: 0.0075 M	Pump expression threshold
	P*ydfA’-eilA*: 0.02 M	
	P*marR’-eilA*: 0.02 M	

## Supporting Information

Figure S1 **Toxicity of [C_2_mim]Cl to** ***E. coli*** **DH1 upon addition of [C_2_mim]Cl.** Red: 0 mM [C_2_mim]Cl, purple: 50 mM [C_2_mim]Cl, blue: 100 mM [C_2_mim]Cl, green: 150 mM [C_2_mim]Cl, yellow: 200 mM [C_2_mim]Cl, orange: 400 mM [C_2_mim]Cl. Error bars represent standard errors.
(TIF)

Figure S2 **Toxicity of pP** ***lacUV5-eilA*** **construct in** ***E. coli*** **DH10B with increasing IPTG concentrations.** Expression of the pump was induced by different concentrations of IPTG. Dark blue: 0 µM IPTG, red: 10 µM IPTG, green: 50 µM IPTG, purple: 100 µM IPTG, light blue: 200 µM IPTG, orange: 300 µM IPTG.
(TIF)

Figure S3 **Model fit to experimental data for growth in the absence of inhibitors.** Blue dots: Experimental data, error bars represent standard error, light Blue: Simulated growth curve.
(EPS)

Figure S4 **Final biomass concentration as a function of IL concentration.** Blue asterisk: experimental data, red: Simulated final values.
(EPS)

Figure S5 **Simulation of toxicity of pP** ***lacUV5-eilA*** **and no IL present.** Blue: α_p_ = 0, red: α_p_ = 0.05, green: α_p_ = 0.1, purple: α_p_ = 0.2, light blue: α_p_ = 0.3.
(EPS)

Table S1 Microarray results after addition of 150 mM [C_2_mim]Cl to exponentially growing *E. coli* cells. Only genes with a change in expression of three-fold or higher and a p-value<0.05 at the 30 min time-point are listed.
(XLSX)

Table S2 Primers used in this study.
(XLSX)
