# Supplementary figures and images for: Development of a Native Escherichia coli Induction System for Ionic Liquid Tolerance
Source: PLoS One. 2014 Jul 1;9(7):e101115. doi: 10.1371/journal.pone.0101115 (PMC4077768; doi:10.1371/journal.pone.0101115)

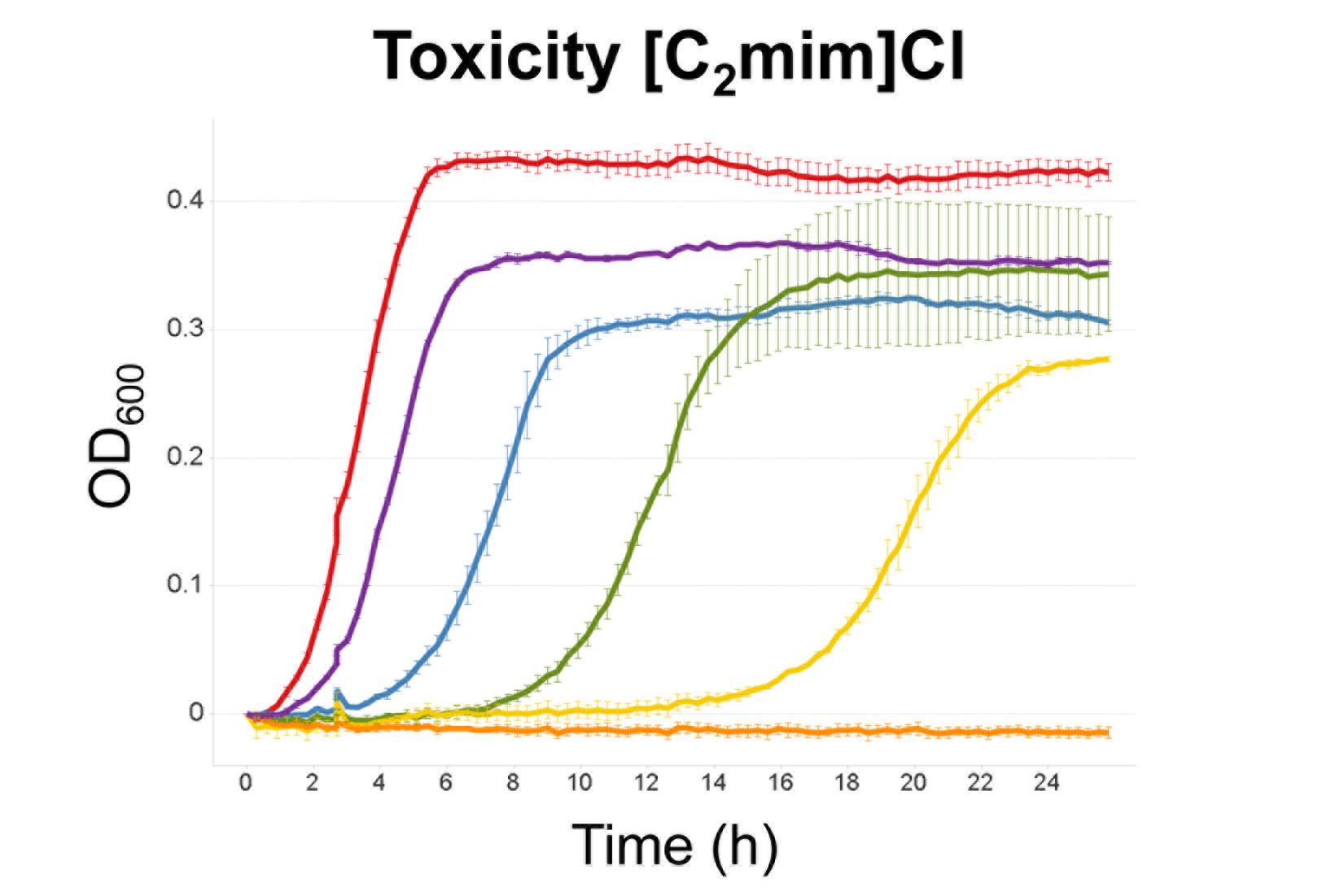

Supplement: Figure S1 — Toxicity of [C2mim]Cl to E. coli DH1 upon addition of [C2mim]Cl. Red: 0 mM [C2mim]Cl, purple: 50 mM [C2mim]Cl, blue: 100 mM [C2mim]Cl, green: 150 mM [C2mim]Cl, yellow: 200 mM [C2mim]Cl, orange: 400 mM [C2mim]Cl. Error bars represent standard errors. (TIF) [file pone.0101115.s001.tif]

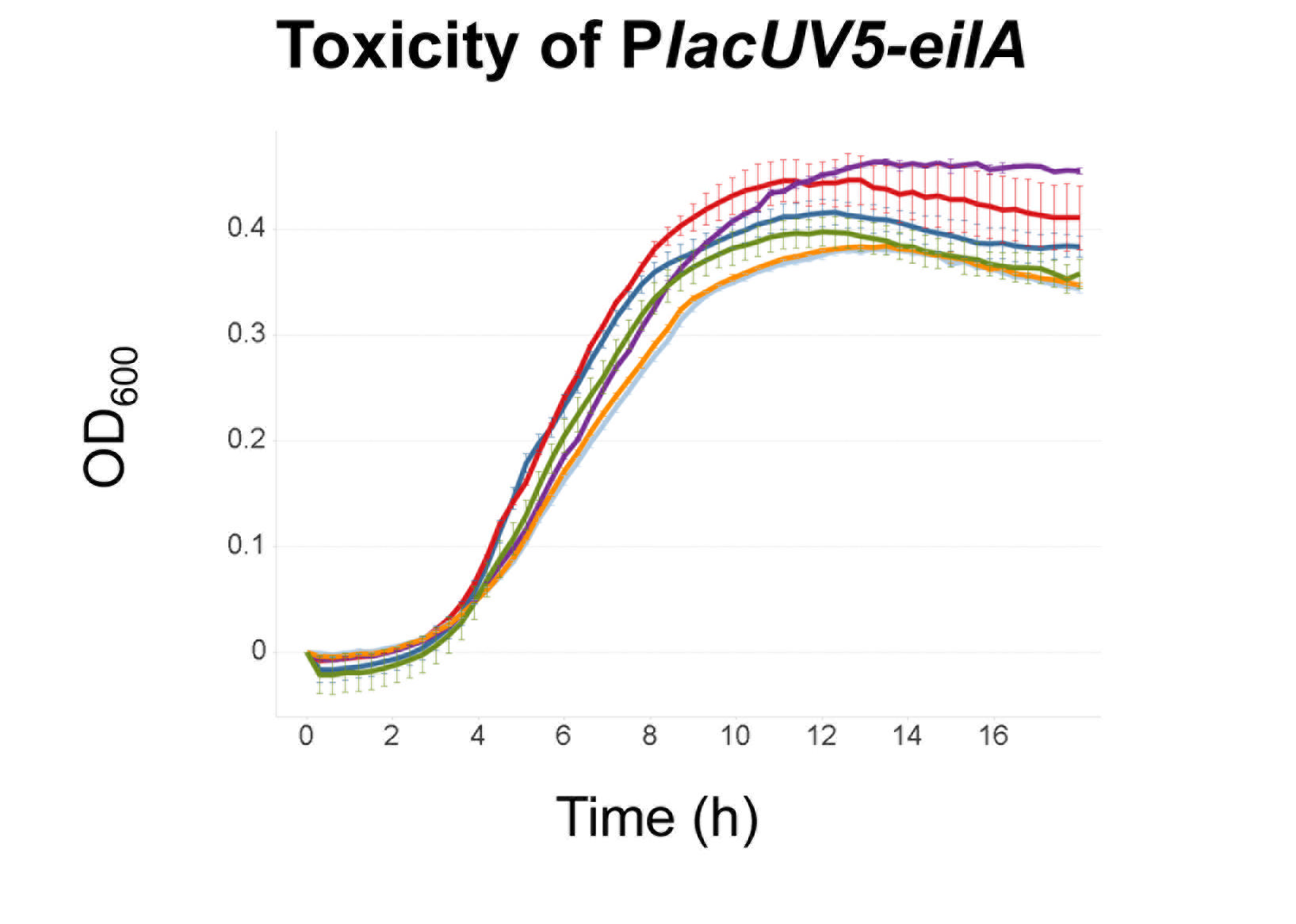

Supplement: Figure S2 — Toxicity of pP lacUV5-eilA construct in E. coli DH10B with increasing IPTG concentrations. Expression of the pump was induced by different concentrations of IPTG. Dark blue: 0 µM IPTG, red: 10 µM IPTG, green: 50 µM IPTG, purple: 100 µM IPTG, light blue: 200 µM IPTG, orange: 300 µM IPTG. (TIF) [file pone.0101115.s002.tif]
